# Highly Sensitive Person: Idiomizing Distress in Pandemic-Era Tokyo

**DOI:** 10.1007/s11013-026-10002-7

**Published:** 2026-07-25

**Authors:** Ramsey Ismail

**Affiliations:** University of California San Diego, La Jolla, USA

**Keywords:** Idioms of distress, Psychiatric legibility, Pandemic mental health

## Abstract

How does an English-language pop-psychology term become a commonly used mental health idiom in Japan, decades after its coinage? Based on twenty months of ethnographic fieldwork in Tokyo at *Raku-no-Kai*, a nonprofit supporting people navigating social withdrawal and related distress, this article traces the local uptake of “Highly Sensitive Person” (HSP) as a vocabulary for narrating vulnerability in everyday life. I argue that HSP functions as an emergent idiom of distress (Nichter, 1981, 2010): a flexible, morally workable language (Myers, 2015) through which people make sensitivity speakable, and at times care-able, without fully submitting to psychiatric diagnosis. Situating HSP within the infrastructures that enabled its spread—digital platforms, self-help publishing, and therapeutic media—I trace the conditions under which sensitivity became newly available as a personal disposition and socially legible form of suffering. The COVID-19 pandemic intensified this process by creating a rare public moment in which strain, withdrawal, and emotional overwhelm became broadly discussable rather than privately borne. Yet HSP’s very looseness also produces friction in clinical encounters, where it can draw people into care while failing to translate cleanly into diagnostic categories. In tracing HSP across peer spaces, media circuits, and the clinic, this article contributes to anthropological debates on idioms of distress, psychiatric legibility, and the contemporary social lives of psychological categories.

## Introduction

How does an English-language pop-psychology term become a commonly used mental health idiom in Japan, decades after its coinage? Building on earlier ethnographic work with *hikikomori* (long-term social withdrawal) exploring the tension between individual adaptation and institutional rigidity (Ismail, 2020), I examine how the idiom of HSP (highly sensitive person) enables people to narrate vulnerability amidst cultural expectations of endurance or composure. I argue that amid a limited public vocabulary for mental health, the growing availability of psychological discourse online, and the intensified attention to distress during the COVID-19 pandemic, HSP became a compelling experiment in how sensitivity could be named, shared, and socially recognized.

These dynamics came into focus during twenty months of fieldwork in Tokyo, where I repeatedly encountered HSP not as a diagnostic claim but as a practical vocabulary for making discomfort legible. For fifteen months, I attended twice weekly meetings at *Raku-no-Kai*’s Aodori Café, a nonprofit community space where people navigating social withdrawal, isolation, and related distress gathered to talk, drink coffee, and exchange strategies for getting through daily life. As an institution, *Raku-no-Kai* occupies an intermediary position between clinical psychiatry and informal peer support, offering low-threshold social contact without requiring diagnosis.

The Aodori Café was an intimate space. On any given afternoon, there were rarely more than eight of us present—myself, the organizers, and a small rotating group of participants—and on some visits only two or three others would show. Those who attended ranged in age from their twenties to their fifties, and the group felt roughly evenly split between men and women, though numbers were too small for this to be more than an impression. That said, HSP as an active self-description appeared to skew toward women. Men were present, but fewer seemed to claim the label openly—those who may have identified with it tended to be quieter about it, suggesting that the gendered stakes of claiming sensitivity publicly were not evenly distributed. In a social context where vulnerability already carries moral risk, the additional burden of performing sensitivity as a man may have made the term less readily available as a communicative resource. Generationally, HSP also skewed younger—though not exclusively so, as Matsuno-san’s case makes clear. The term seemed most fluently adopted by those in their twenties and thirties, for whom online self-help culture and social media were primary sites of mental health discourse. This may partly explain the clinical friction documented later in this article: Physicians of an older generation encountering a pop-psychological category that had circulated largely outside institutional medicine, arriving in their offices already fully formed as a self-description.

Participation was neither stable nor linear: some people came regularly for several months, became familiar presences, and then stopped appearing without explanation—a pattern that itself spoke to the rhythms of withdrawal and re-engagement that brought people to Raku-no-Kai in the first place.

Drawing on scholarship on idioms of distress as culturally mediated socially meaningful forms of communication (Jenkins, 2014; Kaiser & Weaver, 2019 ; Nichter, 1981), I approach “Highly Sensitive Person” (HSP) not simply as a psychological label but as a relational idiom that mediates how distress becomes audible, actionable, and perhaps most importantly, socially acceptable.

This project sits at the intersection of popular psychology and clinical ambivalence, where the boundary between biomedical authority and self-help is continually negotiated in everyday life. In Japan, this negotiation has been sharpened by the growth of patient-centered and peer-based knowledge practices (tōjisha kenkyū), which treat lived experience as a form of expertise and push back against psychiatry’s historically dominant explanatory frames (Ishihara, 2015; Kitanaka & Ayaya, 2023; McCurry, 2018; Tsuboi, 2023)

## Highly Sensitive People: Matsuno and Asano

In a small, humid building on the eastern side of Tokyo, the office of *Raku-no-Kai* transformed twice a week into the Aodori Café, a social space where *hikikomori* and others facing similar struggles gathered to talk, drink coffee, and—most importantly—experiment with new ways of naming their distress. One day in March 2022, I sat at a table divided by thin plexiglass panels. Everyone wore masks. Conversation came through cloth and plastic, muffled but constant.

Matsuno-san, a forty-six-year-old woman, leaned forward and explained, her voice muffled behind her mask: “I’m not quite a *hikikomori*. I have a developmental disability. I’m also a highly sensitive personality type, so I couldn’t handle pressure or loud noises and lights at work. I was always really sensitive and had trouble in school, but I thought I was just a bad person.”

From across the plexiglass, Asano-san—a younger woman in her thirties who described herself as *hikikomori*—nodded fervently and chimed in, almost cheerfully: “I’m HSP too—and I have PTSD.” What struck me immediately was the affect: The stark cheerfulness with which these women expressed what were, on paper, distressing experiences. Their conversation took off at a pace too fast to follow, marked by bursts of laughter and recognition. They compared notes about being quiet kids in class, not liking loud noises, and feeling overwhelmed in big crowds. Rush-hour trains, they agreed, were especially big offenders. In sharing those labels, an instant bond had formed.

Neither woman fit the stereotypical image of *hikikomori*—individuals shut in for years at a time, barely leaving their rooms—nor did they seem especially “sensitive.” I often found myself confused in moments like this: how could people be this many categories at once, truly? And were they actually diagnosed? Yet to question the accuracy of the term would be to miss its power. In this setting, what mattered was not diagnostic precision but the social and moral room that “HSP” created: a vocabulary for self-description that made sensitivity intelligible and socially defensible.

Their chosen term, HSP, is not a clinical diagnosis. Rather, it functions as an idiom of distress (Nichter, 1981, 2010): a culturally resonant register through which people articulate everyday forms of suffering, find community, and negotiate legitimacy. In Japan, where overt expressions of vulnerability can be morally risky, HSP offers a vocabulary that is often more *morally workable* than direct complaint—though, as I show later, it can also be read as self-indulgent, immature, or simply “not real.” For Matsuno-san, however, the term translated pain into a language of attunement.

Matsuno-san returned to the moment she first heard the term. “At first I thought I was a *hikikomori*,” she said. “But that wasn’t necessarily true. At first I thought that word helped me understand, but actually HSP helps me more.” She stirred her coffee in between thoughts, and then said something I heard echoed many times, both in and out of *Raku-no-Kai.* “When people asked me how I was, I was supposed to respond that I was fine *(hai, genki)*. But I wasn’t well, and I just thought something was wrong with me because I wasn’t. The doctor didn’t agree with me when I went, but there’s a real word for me. I’m HSP, and that explains it.”

I made a mental note about comorbidity—symptoms versus illnesses—and about the totality of explanation the word seemed to provide. But I also recognized that the word offered Matsuno-san something she had lacked for most of her life: a register for describing discomforts that had previously been treated as moral failings. Against a social dynamic that demanded composure, *Raku-no-Kai* provided a space, and the term provided the words for Matsuno-san to be sensitive in an insensitive world.^1^

These exchanges suggest that HSP works less as a diagnostic claim than as what Nichter (1981, 2010) calls an idiom of distress—a flexible language through which suffering becomes expressible and thus actionable. Within the everyday talk of Aodori Café, HSP did not operate as a clinical category or a moral stance, but as a communicative resource. It rendered experience discussable in a setting where explicit complaint remains fraught.

HSP can be situated alongside a broader Japanese landscape of everyday diagnostic terms—komyushō, adult children, *dokuoya* among others—that circulate in media and public discourse as ways of making suffering speakable without formal diagnosis, a point I return to later. In what follows, I trace how HSP became a morally workable (Myers, 2015) way to name distress in pandemic-era Tokyo—first by situating it as an idiom of distress, and then by following its circulation across peer spaces, digital media, and clinical encounters.

## Idioms of Distress

Drawing on scholarship on idioms of distress as culturally mediated forms of communication (Jenkins, 2014; Kaiser & Weaver, 2019; Nichter, 1981, 2010), I approach “Highly Sensitive Person” (HSP) not simply as a psychological label but as a relational idiom—one that mediates how distress becomes audible, workable, and, perhaps most importantly, socially acceptable. Idioms of distress matter not because they describe suffering more “accurately” than biomedical diagnoses, but because they show how people communicate suffering under specific moral and institutional conditions: what kinds of complaints are allowed, what kinds are stigmatized, and what kinds can be voiced without social penalty. In this sense, HSP functions as a *non-pathologizing idiom*: a category soft enough to circulate widely, but stable enough to organize experience and make psychic difference speakable—precisely because it requires no institutional confirmation and carries none of the stigma of formal diagnosis.

At the Aodori Café, this meant that HSP could be taken up as a shared vocabulary—one that made discomfort speakable without demanding either clinical proof or moral confession.

In Nichter’s original formulation, idioms of distress offered a way to study culturally specific expressions of suffering without collapsing them into reified syndromes (Nichter, 1981). Later work has emphasized how such idioms can function as pragmatic tools: They can make distress intelligible to others, facilitate recognition, and help people negotiate care—whether that care takes the form of treatment, accommodation, or simply a less morally punishing narrative of the self (Jenkins, 2014; Nichter, 2010). They translate diffuse frustration—about work, family, obligation, and exhaustion—into a culturally resonant form that others can grasp.

And yet, the appeal of idioms of distress lies partly in their looseness: They circulate without requiring institutional confirmation. This flexibility is precisely what makes them powerful, but it also produces an analytical tension. As Kaiser and Weaver note, idioms provide a way to analyze distress without resorting to categorization and reification; yet in practice, once such idioms travel across clinical, bureaucratic, and media infrastructures, they are often pulled back toward precisely those forms. The boundary between “idiom” and “diagnosis” is therefore not fixed. It is actively negotiated in interaction—between people seeking recognition, professionals tasked with classification, and media industries that profit from naming.

HSP in Japan, I argue, occupies this unstable space. It is not a psychiatric diagnosis, yet it borrows the legitimacy of psychology. In practice, it operated less like a diagnosis than like a socially usable approximation of one. It offers recognition without requiring formal diagnosis, and it allows distress to be spoken without fully stepping into the stigmatized space of mental illness. In this sense, HSP functions as a non-pathologizing idiom: a category soft enough to circulate widely, but stable enough to reorganize biography and render psychic difference narratable. What follows traces how this idiom moved—through peer spaces and clinical encounters—and what kinds of recognition and friction it made possible along the way.”

## HSP—Origins, Circulation, Ecology

HSP did not circulate in Japan simply through translation, but through infrastructures—publishers, broadcasters, digital platforms, and the *iyashi* economy—that repackaged a global pop-psychological concept into a locally usable vocabulary of distress.

Despite its medical seeming etymology, “Highly Sensitive Person” (HSP) originates not in psychiatry but in the borderlands between psychology and self-help. In the mid-1990s, American psychologist Elaine N. Aron’s book “The Highly Sensitive Person: How to Thrive When the World Overwhelms You” (1996) introduced HSP as heightened “sensory processing sensitivity,”^2^ followed by a JPSP article (Aron & Aron, 1997) estimating that roughly 15–20 percent of people exhibit this trait. Marketed through best-selling books, workshops, and online quizzes, HSP entered popular psychology circuits where clinical authority and lifestyle coaching overlapped. The term’s success reflected the late modern convergence of therapeutic culture and neoliberal subjectivity—the promise that self-knowledge could yield self-management.

When HSP arrived in Japan in the early 2010s, it did so through this hybrid infrastructure. Japanese translations of Aron’s work were followed by a proliferation of domestic adaptations blending global psychology with local moral sensibilities. Publishers marketed the concept especially toward women and parents of “sensitive children,” resonating with longstanding anxieties surrounding social and interpersonal issues such as *futōkō* (school non-attendance) and *hikikomori* (social withdrawal). By 2015, HSP appeared regularly in wellness sections of major bookstores, often adjacent to guides on communication or mindfulness. Television programs and online quizzes popularized it further, presenting HSP as a personality type that could explain everyday difficulties with crowds, noise, or interpersonal pressure (NHK, 2019; Kondo, 2021). Through these translations, sensitivity was understood as something one could both discover and manage.

This domestication of HSP exemplifies how idioms of distress travel not only linguistically but infrastructurally. Publishers, wellness industries, and digital platforms act as intermediaries that reconfigure psychological knowledge for local audiences (Lock, 2013; Nguyen, 2010; Yergeau, 2018; Zhan, 2009). More broadly, this is a familiar problem in medical anthropology: categories of suffering rarely circulate on their own, but move through political, commercial, institutional, and media infrastructures that shape what counts as legitimate distress, who can claim it, and what forms of care become thinkable (Briggs & Hallin, 2016; Lakoff, 2005; Martin, 2007; Nguyen, 2010; Rose, 2007; Young, 1995).

In Japan, this process unfolded within what scholars call the *iyashi*—or “healing”—economy: a commercial field that promises emotional restoration through lifestyle products, books, and media (Allison, 2013). HSP thus circulated as both self-diagnosis and commodity, a label that offered comfort whose alignment with consumerist ideals of self-care makes it an easy sell.

This circulation was further catalyzed by the COVID-19 pandemic, which in Japan as elsewhere, intensified public conversations about stress, isolation, and vulnerability. During 2020–2022, Japanese publishers released numerous new HSP titles, YouTube therapists hosted live “sensitivity” sessions, and major broadcasters (e.g., NHK) featured segments presenting HSP as a relatable way to describe pandemic-era overwhelm. Clinicians were often skeptical, but its everyday uptake revealed something more consequential: people sought in HSP a socially acceptable language for distress. Under home restraint, when daily life slowed, and time alone expanded, sensitivity became a topic of conversation—and an easy explanation for an up till then life full of tension.^3^

As one young woman at *Raku-no-Kai* told me, “When I heard that term, I thought: maybe I’m not weak, I’m just sensitive,” allowing her to recode fragility as a mode of attunement, and reinterpret her own experience of empathy. Rather than rejecting expectations of composure and resilience, the label softened them—translating endurance into a more bearable sensitivity.^4^ It is worth clarifying that in using the language of attunement here, I am not claiming that HSP produces interpersonal connection or therapeutic reciprocity in any clinical sense. Rather, I follow my interlocutors in observing how HSP functioned as an organizing vocabulary—one that brought together what had previously been experienced as disparate, unrelated difficulties and gave them a common name. Whether that coalescing is understood as attunement, sensitivity, or simply recognition, what mattered to those who used the term was less its psychological precision than its capacity to make a scattered set of experiences feel coherent.

HSP’s Japanese context mirrors what Hacking (1999) calls the “looping effects” of humankinds: once named, categories reshape those who use them, are reshaped by those who use them, and continue to change in an iterative process. Yet this looping does not unfold in a vacuum.

It is mediated by institutions of translation and consumption—publishers, broadcasters, and digital platforms—that determine which forms of distress become audible. Though Hacking focused on recursive loops specifically, the HSP boom demonstrates how idioms of distress simultaneously circulate through personal, institutional, and transnational feedback loops: Theories become commodities, commodities become identities, and identities return to the clinic demanding recognition. What began as an American self-help term thus became a communicative resource for pandemic-era Japan—both the term and the people that use it remain changed in the social afterlife of its adoption.

Married with a proliferation of HSP-related media, the interplay between person and category, and the general expansion of the term’s use, I argue, created conditions ripe for its sudden ubiquity. HSP in Japan illustrates the formation of an *ecological niche* (Hacking, 1998, 2007): a social environment dense with moral, institutional, and communicative affordances that allow certain psychological categories to take root. In this sense, HSP did not simply transform individuals after its naming; it flourished because pandemic conditions in Japan provided the moral and infrastructural terrain for it to make sense.

This public uptake had a recognizable social life beyond *Raku-no-Kai*. In Japanese popular psychology and wellness media, the “HSP / 繊細さん” label consolidated around a visible boom around 2020, closely tied to the mass-market success and media circulation of titles such as *『「繊細さん」の本』* (Takeuchi, 2018). By late 2020, the book’s publisher publicly framed it as a breakout cultural event, reporting sales surpassing 500,000 copies (Asuka Shinsa, 2020). By 2023–2024, the term was also being assessed in explicitly retrospective registers—most notably in a widely circulated booklet that framed “HSPブーム” as an object of critique and clarification (Iimura, 2022), and in professional psychological commentary asking what the “HSP boom” had been and what its consequences were (Asahi, 2023). This niche may also have been time-bound. While HSP was ubiquitous in the spaces I moved through in 2022, colleagues in Tokyo noted that by 2025 the term appeared less frequently in everyday talk.^5^ Taken together, these traces suggest not only the diffusion of a category, but a temporally bounded moment in which “sensitivity” became newly nameable—followed by a phase in which its meanings, misuses, and durability became topics of public and professional reckoning.

To this day, many clinicians in Japan remain skeptical of HSP’s validity and treat it as a pop-psychological fad rather than a diagnostic category. On the one hand, some—such as psychologist Iimura Shūhei (2023)—warn that the boom enables dubious credentialing and diagnostic entrepreneurship; on the other, psychiatrist Matsumoto Takuya and sociologist Doi Takayoshi emphasize that even without diagnostic standing, such terms can still function as pragmatic entry points for narrating distress (Asahi Shimbun, 2023, 2021). Either way, its everyday uptake demonstrates how non-diagnostic labels can reshape cultural understandings of distress and provide alternative avenues for self-identification.

HSP also belongs to a broader Japanese ecology of everyday diagnostic labels—such as コミュ障 (*komyushō*, roughly “bad at communication”), “adult children,” and 毒親 (*dokuoya*, “toxic parents”)—alongside terms with clearer clinical standing such as 発達障害 (*hattatsu shōgai*)—through which distress is narrativized, stigma is managed, and moral personhood is renegotiated in public life (Borovoy, 2008; Hacking, 2007). These terms circulate in media and everyday conversation as semi-clinical vocabularies: They borrow the legitimacy of psychology while remaining available for casual self-description, explanation, and social positioning. As anthropologist Amy Borovoy (2008) argues in her account of “mainstreaming” Tokyo’s youths, such vernacular categories do not merely describe suffering; they help reorganize the moral terms under which marginal lives become recognizable, discussable, and potentially reintegrable.

## Pandemic + Self-restraint (Why Now)

HSP’s resonance in Japan cannot be separated from a moral landscape that prizes composure. The COVID-19 pandemic did not create these expectations, but it intensified them—and newly made them visible as a subjective experience. At *Raku-no-Kai*, participants often joked that this year we were all *hikikomori*. As one man put it, the virus “didn’t care who you are,” producing an uncanny equality of exposure. Fragility, once stigmatized, became newly shareable.

A 2023 government survey found approximately 1.5 million working-age Japanese identifying as social recluses after COVID-19—with roughly one-fifth attributing that status to pandemic pressures (McCurry, 2023). Media outlets noted that even long-term *hikikomori* and formerly active individuals faced similar confinement (Wired, 2021). Although many businesses remained open, public behavior treated restraint as de facto lockdown—millions struggled to adjust. As homes became confinement zones, many looked to a formerly marginalized demographic—*hikikomori*—for codes of survival, and for a moment it seemed they had shifted from pariahs to inadvertent mentors (Kyodo News, 2020). In this moment, everyday attitudes toward mental health—including *hikikomori*—stood to be profoundly reconfigured (Kato et al., 2020; Takahashi, 2020; YahooJapan, 2020).

Yet this new shareability also reshaped how people attended to their own mental states—making ordinary vulnerability feel newly collective.

HSP’s uptake in Japan intensified during COVID-19 not because the pandemic produced sensitivity, but because it made the labor of composure newly visible. The virus did not simply isolate people; it reorganized everyday life around the ethics of restraint. In Tokyo, *jishuku*—voluntary self-restraint—became less a policy than a mood: A quiet civic demand to be careful, not to impose, not to take up too much space. Masks, plexiglass, shortened conversations, and the choreography of avoiding others on trains and sidewalks turned attunement into a kind of moral practice. To live through the pandemic was to constantly monitor one’s own presence—breath, voice, distance—as if ordinary social life had become newly fragile. This mood of restraint was so pervasive that even in summer—when temperatures regularly climbed above 35 degrees Celsius and stayed there—signs encouraging people to remove their masks if they feared heat stroke appeared in building walkways and on trains.

For many of my interlocutors, HSP resonated in this moment because it offered a language for something they had long felt but rarely been able to name: that modern life already required them to endure too much, and that “getting through it” came at a cost. The pandemic did not invent overstimulation or exhaustion, but it made them newly discussable. As a result, HSP helped translate diffuse strain into a socially legible form—one that could be voiced without requiring the heavier moral and institutional consequences of psychiatric diagnosis.

This revaluation of a previously well-known sensitivity transformed the terrain of mental health my interlocutors faced—as both longstanding *hikikomori* and successful businessmen and women with no prior history of mental health conditions expressed that mental health had gone from something that affected others “out there” to a local problem everyone had to face. Researchers suggest that during COVID-19 the line between enduring withdrawal and enforced isolation blurred, rendering a differentiation between pathological and “non-pathological *hikikomori*” newly unstable (Kato et al., 2024). What had been a marginalized condition now resonated with the generalized strain of a society turned inward. An insensitive virus, paradoxically, created a space and time for sensitivity—making removal from social participation not only visible but expected, creating a viable “ecological niche” (Hacking, 1999) for Highly Sensitive Personality.

HSP’s reach extended well beyond support spaces like Raku-no-Kai. In the spring of 2022, a longtime friend mentioned that a colleague of hers—a woman in her late thirties who worked at a major Tokyo airport—had attempted to invoke her HSP as grounds for requesting additional leave during the pandemic to visit her family in another prefecture. The woman had come to understand herself as HSP over the course of the previous year, largely through online resources and the wave of self-help publishing that accompanied the pandemic. She presented the term to her manager as an explanation for why the sustained disruption to her routines and the emotional demands of working in a high-traffic public environment during COVID-19 had taken an unusual toll. Her manager was unmoved. HSP, he told her, was not a recognized condition and did not constitute grounds for special accommodation. She was eventually let go. My friend, recounting this, was matter-of-fact: “Yeah, I’d heard of it,” she said, “but it was tricky.” She suspected the HSP request had soured the manager’s view of her colleague—that invoking it had read less as a genuine disclosure of difficulty than as an attempt to leverage a fashionable label for personal benefit.

What struck me about this account was less the manager’s refusal—predictable, given HSP’s lack of clinical standing—than what the attempt revealed about HSP’s ambivalent social life. The woman had reached for HSP as though it were a legible institutional claim, a vocabulary sufficient to translate private distress into a form that bureaucratic authority might recognize and accommodate. That it failed—and that its failure carried professional consequences—exposed something important about the limits of non-pathologizing idioms. HSP travels well in peer spaces and self-help circuits precisely because it makes no formal diagnostic claim: It is soft enough to share, gentle enough to try on, morally workable in settings that do not demand proof. But in contexts where recognition requires institutional authorization—a manager’s office, a human resources form, a clinical chart—that same looseness becomes a liability, and can even become evidence of bad faith. The term that renders suffering speakable among peers cannot, in these moments, render it actionable in the ways that might actually change the conditions producing it. Worse, in the wrong hands and the wrong context, it can be read as the very thing it was designed to resist: “a performance of fragility rather than an honest account of it.”

HSP thus performs cultural translation twice over. It imports a global psychology of personality while localizing it within Japan’s longstanding ethic of restraint. Rather than rejecting expectations of composure and resilience, HSP offered a way of speaking their effects—rendering sensitivity not as failure, but as the lived cost of getting through.

Yet precisely because HSP functioned as recognition without diagnosis, it often became difficult to translate in clinical settings—where distress must be made commensurable with standardized categories in order to count as treatable.

## From Diagnosis to Identity (and Back Again)

The circulation of HSP in Japan during the pandemic thus reflects a wider shift in how psychological and developmental categories move between medical diagnosis and personal identity (Navon & Eyal, 2016; Navon, 2019; Rosenberg, 2006). In recent decades, conditions such as autism and ADHD have undergone similar transformations, as diagnostic labels have become resources for self-understanding, community, and activism (Bagatell, 2007; Eyal et al., 2010). As Navon (2019) argues in his study of the global autism field, the boundary between disorder and difference increasingly depends on local moral and institutional contexts—on what kinds of difference can be recognized without stigma, and to what ends those forms of recognition are mobilized.

In Japan, autism and ADHD have long occupied ambiguous terrain. As the collection by Carolyn Stevens (2013) notes, public awareness has grown, but the line between developmental disability/impairment (*hattatsu shōgai*) and mental illness (*seishin shōgai*) remains porous. Individuals, their parents, and educators often mobilize these labels strategically: to access accommodations, to resist blame, or to frame a child’s behavior as biological rather than moral failure. For adults, these same categories sometimes offer retrospective coherence. The English acronyms (ADHD, ASD, HSP), rather than their longer Japanese diagnostic terms, can also function as a shorthand that feels portable, modern, and institutionally backed—even when clinical standing is ambiguous. Online communities for “late-diagnosed” individuals—particularly women—echo Matsuno’s narrative: relief at discovering that longstanding exhaustion or misfit is not a personal defect but a named pattern with seeming origins in science. HSP circulates through these same networks, often adjacent to ADHD and autism discussions, yet it occupies an even looser and less medical register.

Unlike ADHD or autism, HSP circulated largely outside formal pathways of diagnosis and accommodation. Its power lies in its elasticity: It could be taken seriously while remaining socially usable. This flexibility allows people to adopt the term selectively, to use it as vocabulary rather than verdict. For some, HSP functions as an entry point into the world of mental health discourse; for others, it offers an alternative to the pathologizing tone of diagnosis. Its appeal lies in the way it borrows the legitimacy of psychology while remaining safely in the realm of personality and temperament.

Comparisons with *taijin kyōfushō*—the social anxiety syndrome identified in Japan in the mid-twentieth century—make this point clearer. *Taijin kyōfushō* expressed distress through the fear of offending or burdening others and was once treated as a culturally specific disorder (Kirmayer & Sartorius, 2007; Kitanaka, 2012). In contemporary Japan, its vocabulary persists in casual use but rarely carries clinical weight. HSP, by contrast, reverses the direction of that concern: rather than fearing the impact of one’s behavior on others, HSP foregrounds one’s sensitivity to the environment—noise, emotion, pressure—as the problem. Both syndromes articulate the tension between relational obligation and psychic limits, yet they differ in moral geometry. *Taijin kyōfushō* framed suffering as over-responsibility to others; HSP reframes it as overstimulation by others. HSP also belongs to a longer Japanese history of idioms that rise, circulate, and fade—one earlier example being neurasthenia (*shinkei suijaku*), which once offered a widely intelligible language for exhaustion and nervous depletion, before losing traction as psychiatric and popular vocabularies shifted (Kitanaka, 2012)

**Fig. 1:**
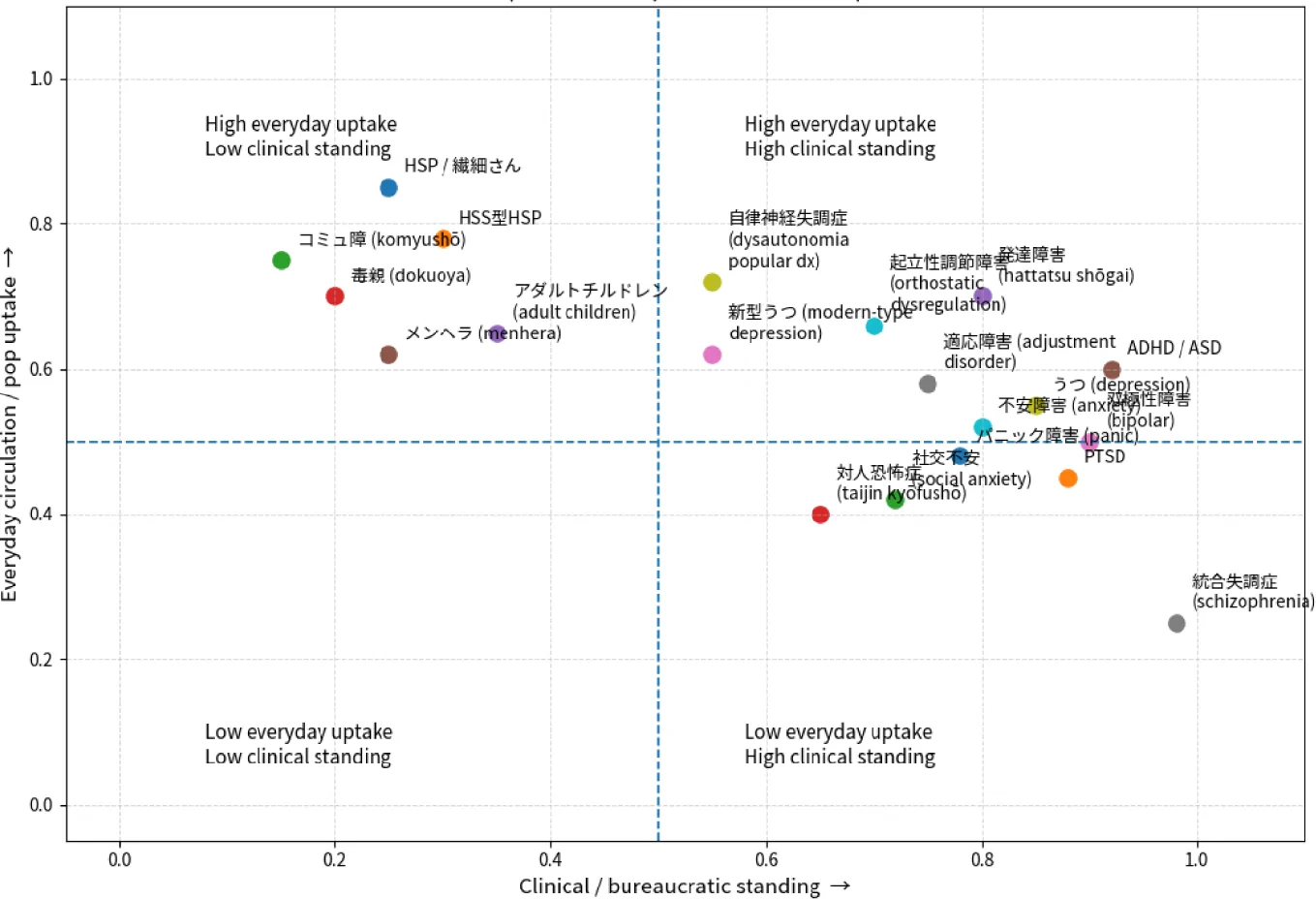
Map of Clinical and Vernacular Labels in Contemporary Japan (Illustrative, Not Exhaustive)

At the same time, HSP circulates within a broader ecology of everyday diagnostic labels—コミュ障 (komyushō), “adult children,” 毒親 (dokuoya), and others—that help people narrate distress in ways that do not require clinical confirmation. These terms often operate retrospectively, reorganizing a life story around a newly available explanation, and reclassifying what had once been felt as weakness, immaturity, or personal failure. Figure 1 offers a rough map of how such labels cluster across axes of clinical/bureaucratic standing and everyday circulation.

### Shotaro’s Skepticism

And yet, despite this boom, HSP was not universally embraced. Outside spaces like Aodori Café—where participants were unusually willing to experiment with new vocabularies of distress—the term could be met with skepticism, irritation, or refusal.

Shōtarō, a working professional in his thirties whom I met through my homestay mother’s bar, voiced clear skepticism when I brought up the term. Longstanding sleep problems and a long-distance relationship going through a rough patch kept him up late at night, where boredom and frustration led him into a deluge of YouTube videos, TikToks, and Instagram reels about mental health. Unlike Matsuno-san, Shōtarō was not an instant believer in HSP. He admitted that before his own struggles with depression and anxiety, he had never heard the term—and did not take it seriously.

“Those short clips online—even though I didn’t believe them—were kind of dangerous,” he explained. “They’re structured to make you think you’re HSP. That’s dangerous for people who are desperate to understand themselves. But with how long it takes to see a doctor, sometimes that’s all they have. I had to wait over a month to get an appointment.”

Shōtarō’s concern echoed broader critiques of psychiatrization: the spread of psychiatric categories into ever-wider arenas of life, where they become resources for identity and belonging well beyond clinical settings (Rose, 2007). As Deborah Lupton (2017) argues, social media platforms increasingly function as sites of self-diagnosis and self-education, where psychological labels are packaged into easily shareable lists, quizzes, and short-form narratives. These formats do not simply circulate categories; they invite users to inhabit them, offering immediate explanatory relief in moments when professional care is inaccessible, stigmatized, or delayed.

3For Shōtarō, the danger lay less in the existence of the category than in its overgeneralization.

“Isn’t it just a descriptor?” he asked. “A symptom, not an end result. It might be a good tool for recognition, and maybe it gives people some insight. But online, the criteria are so broad that anyone could think they’re HSP. Content creators do this on purpose; in order to maintain engagement, the criteria have to be so general that anyone could find the page and find a reason to stay on it.” He paused, and then added a worry that was less about misdiagnosis than about selfhood. “And what if people start acting more HSP just because they’ve found the label, or stop trying to act otherwise? People spend so much time on their phones now. Algorithms cater your content to search terms so easily—how could you not find these videos?”

Shōtarō expressed particular concern for younger Japanese people—“this seems really dangerous for youth”—who increasingly rely on their phones as a primary interface through which to make sense of the world around them. What he flagged was not only the possibility of misrecognition but the generative force of the category itself: The way a label can reorganize attention, biography, and social expectation once it becomes available.

Anthropologists have long noted how medical and psychological classifications do not merely name suffering but can actively reshape how people experience and narrate it. Joseph Dumit (2004), for instance, describes how biomedical images and categories encourage people to “live as if” a diagnosis were already true, reorganizing everyday life around a classificatory promise. Emily Martin’s (2007) work on bipolar disorder similarly shows how diagnostic categories can become frameworks through which people interpret moods, relationships, and futures—sometimes in ways that feel clarifying, and sometimes in ways that feel constraining. Shōtarō’s skepticism, in other words, was not simply about accuracy. It was about the possibility that HSP might multiply distress by pulling people into inhabiting the category, precisely because it was the most accessible language available while waiting for—or actively avoiding—professional care.

### From a Recovered *Hikikomori*

If Shōtarō worried about the overreach of pop-psychological categories online, Nakamura-san emphasized something else: The pragmatic relief such terms can offer in the absence of diagnosis, accommodation, or socially recognized explanations.

As a frequent attendee at Aodori Café, Nakamura-san had followed HSP’s popularization closely. He recognized that the term had acquired a casual, almost ubiquitous valence, often used in tandem with *hikikomori*. Having heard these words numerous times, and both folks with and without mental health categories attached to them use these words, I brought them up in a capsule art activity planning session with Nakamura-san, a former *hikikomori* who now worked as a recruiter, helping former *hikikomori* find new jobs. Gainfully employed, married, and with two children, Nakamura-san aimed to start an NPO that combined sustainability and mental health, recycling the raw materials from Japanese capsules for toymaking activities for *hikikomori*, social anxiety, and developmental disabilities, in hopes that others could find their way to some sort of recovery.

As Nakamura-san told me, “it did not matter whether the word was an exact fit.” In the absence of formal diagnoses, idioms like HSP—circulating through the internet, cafés, and informal support groups—communicated commensurable experiences and “allowed participants to recognize themselves in one another.” *(otagai ni mitometekureru*) After all, no one had ever formally diagnosed him as *hikikomori*. “These words,” he explained, “provide an excuse—or maybe an avenue—to fail or to stop doing, at competitive jobs, school, or otherwise in a society that doesn’t allow much room for failure. And then, when you go to the café or you’re online, you can say you have HSP, or PTSD, and maybe someone else will too.”

He continued: “The limit for what you can be in Japan is so narrow. So much is defined by work, or school, or some recognizable social status. Outside of those avenues, it’s hard to have an identifier, and for others to understand you—and in turn to understand yourself.” Nakamura-san echoes what sociologist Mary Brinton (2011) describes as Japan’s postindustrial narrowing of “social locations,” where institutional pathways linking education, employment, and adulthood have eroded, leaving individuals suspended between identities once guaranteed by work and school. In this landscape, categories like HSP offer alternative anchors of self-understanding for those who no longer fit neatly into the structures that once conferred meaning and recognition. Much like Myers-Briggs types in the U.S., these categories feel scientific, offering concise explanations for social and emotional struggles.

Yet, as Nakamura-san noted, people can also come to inhabit them too completely, giving up on other ways of being or changing—a dynamic long recognized in anthropology. Put plainly, people *become* the category that once simply *described* aspects of their behavior, perhaps as a life sentence rather than a descriptor. Precisely for this reason**,** HSP’s usefulness as an idiom of distress—its looseness, portability, and everyday legitimacy—is also what makes it analytically unstable. It offers recognition without diagnosis, but it can also become a template for selfhood.

The point here is not simply that labels reshape subjectivity—a dynamic well described in anthropology and STS (Hacking, 1999; Rose, 2007)—but that classificatory systems have consequences even when they are informal, unstable, or only half-legible to institutions (Bowker & Star, 1999). What matters is why this particular label became so usable, so quickly, in pandemic-era Japan. HSP offered a way to make vulnerability intelligible without incurring the institutional weight of diagnosis, and without requiring that one’s suffering be “serious enough” to count as illness. In that sense, HSP did not merely name distress; it made distress socially shareable and, at times, care-able, even as the clinic remained ambivalent, delayed, or difficult to access.

## Clinics & Commensuration (or a Lack Thereof)

In clinical settings, HSP becomes a site of friction. The same looseness that makes the label so usable in everyday life—portable, morally gentle, and widely shareable—also makes it difficult to translate into diagnostic categories. HSP can draw people into care and help them articulate experience, yet it often fails to become commensurable with the kinds of suffering psychiatry is trained to recognize as treatable. This gap is not only interpretive; it is infrastructural. Clinical encounters require that distress become legible in standardized terms in order to count as treatable.^6^ As Bowker and Starr remind us, classification systems do not simply describe the world—they structure what can count, what can be recognized, and what can be acted upon as real (1999). HSP often fails that test, precisely because it is so socially usable. What emerges is not simply misunderstanding, but a commensuration gap: patients and clinicians working in parallel idioms, both oriented toward relief, but rarely meeting in the same language.

In clinical encounters, patients often brought HSP with them as both biological predisposition and vernacular diagnosis—a coexistence that required constant translation. That translation work, Lock and Nguyen (2010) note, is both linguistic and infrastructural: Clinicians and patients must align different regimes of evidence, authority, and affect. Rather than confusion, Nishijima-sensei’s lamentation in psychiatric settings reflects an improvised attempt to keep care possible amid a lack of shared ground, even if it often failed.

Such frictions are hardly unique to HSP. The effort to turn lived distress into standardized illness, long recognized in anthropological writing (Good, 1994; Lock, 1993; Young, 1995), continues to generate uneven terrain where subjective experience resists full incorporation into clinical form. This lack of commensuration in medical settings is well documented (Kleinman, 1978, 1988; Mol, 2002) and specifically in Japan (Borovoy, 2005; Kitanaka, 2012; Nakamura, 2013)—Junko Kitanaka shows in her ethnography of depression treatment, clinical practice in Japan translates social suffering into psychiatric cures while reconfiguring patients’ self-understandings (Kitanaka, 2012). Patients arrive with a vocabulary grounded in sensitivity and strain; psychiatrists rely on the categorical grammar of the DSM.

### Nishijima-sensei: The Clinical Perspective

It was exactly this lack of supervision that many folks found suspicious. Indeed, the first time I began talking about HSP at an academic conference in March 2021, I was encouraged to move away from the topic. It wasn’t something to take seriously in a clinical context. Eager to understand what was illegitimate about this term that kept popping up, in June 2022, I sat with Nishijima-sensei, a clinician who had attended with me one of the first large post-pandemic seminars for families of *hikikomori*.

Nishijima-sensei worked both in private practice and as an occasional lecturer at a prestigious Japanese university. We sat together at the seminar, and he introduced himself to me, curious about the presence of a tall, light-skinned foreigner at an event usually exclusively attended by Tokyo natives. The seminar had drawn more than sixty attendees—the highest level of participation since the pandemic began, when events regularly drew close to a hundred—and detailed a moving story of a withdrawn *hikikomori* daughter who had reconnected with her father through a community-based approach. Over iced coffee in a cramped *kissaten* heavy with the smell of tobacco, we reflected on the seminar’s success, the fungibility of a recovery story that had required the mobilization of an entire community, and on the challenges posed by new vocabularies like HSP.

I raised the question directly: what did he make of patients introducing themselves as “HSP”? He paused before answering:

“Sure, a lot more people have come to see me using that word. But I can’t treat ‘high sensitivity,’ especially if it’s framed as a personality trait rather than an illness. The problem is that many patients have depression or anxiety—I can treat those. When they talk about being HSP, they are talking about important experiences, but their desire to be treated for this specific thing doesn’t work.” Nishijima’s hesitation echoed a familiar clinical dilemma: what does psychiatry do when a person arrives not with an illness to cure, but with an “inherent” trait to live with—something closer to autism’s contemporary framing as difference than to depression’s framing as disease?

Nishijima’s reflections pointed to the central contradiction in HSP’s everyday life: It could bring people into clinical spaces, but it simultaneously complicated diagnosis and treatment. As discussed earlier, this is precisely the double edge of HSP as a non-pathologizing idiom: It offers recognition without diagnostic consequence, and it is that very non-pathologizing quality—the feature that makes it socially portable and morally gentle—that simultaneously makes it difficult to translate into clinical categories where distress must become commensurable in order to count as treatable. As early as 1988, Arthur Kleinman’s foundational work on explanatory models shows how idioms of distress often lose meaning once translated into clinical categories, which are often subject to their own logics of evidence and intervention. More recent analyses demonstrate that such translation problems persist under digital and pandemic conditions, in which non-professionals increasingly come into contact with medical-sounding language (Briggs & Hallin, 2016; Lupton, 2014a, 2014b). For Nishijima, HSP was neither a diagnosis nor a treatment target. He seemed torn: He acknowledged that the term brought patients to his office, allowing them to articulate vulnerabilities that might otherwise have remained invisible. But simultaneously frustrated, when self-diagnosed patients pushed back on his offer of other possibilities.^7^ This tension—between HSP’s usefulness as an idiom and its fragility as a clinical object—was not unique to Nishijima. As Japanese media commentary on the HSP boom suggests, the category’s rapid uptake has been accompanied by growing public debate over its lack of diagnostic criteria, its ambiguous relationship to psychiatric expertise, and the opportunistic businesses that have emerged around it. As psychologist Iimura Shūhei writes, “there is no clear criterion” by which people can be divided into HSP and non-HSP, even as the term circulates as if it named a stable type (Asahi Shimbun, 2023, 2021).

Patients I spoke with echoed these events and often described going to their doctors armed with the language of HSP, only to be brushed aside. One young woman told me she tried to explain to her physician that she was HSP; he simply moved on, uninterested. Many complained that such dismissals led to delayed diagnoses, untreated conditions, and a deepening mistrust of medical care. On the other hand, Nishijima-sensei lamented that when patients insisted on HSP as their primary identity, he could do little for them: The term lacked diagnostic clarity, and he felt uncertain about what he was actually treating.

Treatment thus falters not through conflict but through the slow erosion of mutual intelligibility. Coupled with a consumerist mindset, under Japan’s national health insurance system, psychiatric care is often accessible in principle, but in practice constrained by brief visits and standardized diagnostic requirements. Patients may arrive expecting recognition and explanation; clinicians may be able to offer treatment only by translating distress into categories that do not quite fit. Both can leave the encounter frustrated: Patients without the resolution they hoped for, and clinicians without a category they can clearly treat.

What emerges then, is less a crisis of definition than a commensuration gap: patients and clinicians working in parallel idioms, both oriented toward relief but rarely meeting in the same language. Yet even in this disagreement, these labels held value. As I learned from the attendees at Aodori Cafe, though they weren’t reliable, they were valid.

### *Raku-no-Kai* as a Moral Laboratory

As such, the Aodori Café functioned as more than a meeting place. It was a space where people could test new ways of speaking about themselves—what Cheryl Mattingly (2014) elsewhere calls a “moral laboratory”. Within Japan’s uneven landscape of psychological education—where idioms of distress have repeatedly risen, circulated, and fallen out of fashion across the twentieth century (Borovoy, 2008; Kitanaka, 2012)—*Raku-no-Kai* became an experimental ground for practice. Conversations moved fluidly between discussions of *hattatsu shōgai* (developmental disability), PTSD, and HSP, not to establish correctness but to search for resonance. The point was not diagnostic precision but recognition. As one participant told me, “This is why it’s an *ibasho*—a place where I can figure out how to just be, without expectations.” What mattered here was not whether any label was clinically correct, but whether it made space for a person to remain in relation—to speak, to hesitate, to be understood—without immediately being measured against the demands of work, family, or treatment.

Against the pandemic backdrop of suspended productivity, proliferating online discourse, and collective isolation, *Raku-no-Kai* became a rare venue for reimagining sensitivity as social rather than selfish to express. Under these overlapping pressures *“Highly Sensitive Person”* began circulating in earnest—conditions ripe for its proliferation. And it was in this ecosystem that I first walked into *Raku-no-Kai*.

## Conclusion

To quote Hacking one last time, “The banal question of whether… illnesses are real is not very interesting. Of course they are real enough for those who have them” (Hacking, 1999). What matters more, then, is what the category *does*. Here, that work is less about psychiatric accuracy than social life: The creation of a space where people can test the meanings of sensitivity in a society that rarely permits it, and the fragile possibilities for understanding and treatment that appear when patients and doctors begin to meet in that space.

This article has traced how “Highly Sensitive Person” became usable in pandemic-era Tokyo as an idiom of distress—one that offered recognition without diagnosis, and a way of making vulnerability shareable without requiring that one’s suffering be “serious enough” to count as illness. Following HSP across peer conversations, online infrastructures, and clinical encounters, I have argued that its power lies less in its psychological validity than in its social portability: the way it can circulate as a morally gentle explanation, stable enough to organize experience but loose enough to avoid the institutional weight of psychiatric categorization.

The dynamics traced here resonate with broader anthropological accounts of how new emotional languages take hold in contexts where biomedical psychiatry has historically dominated—Tomas Matza’s work on the psychotherapeutic turn in post-Soviet Russia, Li Zhang’s account of the inner revolution brought by psychotherapy in urban China, and Allen Tran’s examination of emotional vocabularies in Ho Chi Minh City all document how psychological idioms, imported and domesticated, become resources for navigating precarious social worlds (Matza, 2018; Tran, 2015; Zhang, 2020). HSP in Japan belongs to this wider landscape. Yet this case adds a dimension these accounts do not centrally address: the social power of a resolutely non-pathologizing idiom. Where therapeutic cultures in Russia, China, and Vietnam have tended to move distress toward clinical or quasi-clinical recognition, HSP in pandemic-era Japan worked in the opposite direction—gaining traction unfettered by the diagnostic weight that formal psychiatry would impose. What this case contributes to these comparative conversations is an account of how idioms of distress can be powerful not despite their institutional weightlessness but because of it.

Seen from four vantage points—support spaces, the skeptic, the clinician, and a recovered *hikikomori*—HSP’s social life in pandemic Japan comes into view. For Asano-san and Matsuno-san, the label reorganized childhood memories into a coherent life history, turning discomfort into a socially legible idiom that would have been useful when they were much younger. For Shotaro, HSP signaled the dangers of psychiatrization in the age of social media, where categories are amplified and flattened by algorithms that invite ever-wider self-identification. For Nishijima-sensei, it was both a tool of access and an obstacle to care—valuable for drawing patients into the clinic yet vexing in its lack of diagnostic traction. And for Nakamura-san, a recovered *hikikomori*, HSP carried both the promise of mutual recognition and the risk of becoming a ‘trap’: another promising label with the potential to inhabit too completely.

These ethnographic moments also clarify what I elsewhere describe as *strain*: the friction between conditional rigidity and human adaptability (Ismail, forthcoming). HSP crystallizes that tension, showing how individuals in Tokyo adapted to navigate overlapping crises of recognition, trust, and endurance. In naming sensitivity, they reveal the pressure points of a social order that demanded emotional calibration as both cure and cause. This is the paradox of idiomizing distress: The language that renders suffering legible (such as *HSP)* can also reproduce the structures that sustain it.

As Veena Das (2007) observes, suffering unfolds in the minutiae of everyday life, where new languages of care emerge not as ruptures but as quiet acts of endurance. As I hope to have shown, *Raku-no-Kai* thus constitutes a promising moral laboratory (Mattingly, 2014): a place where people experiment with the meanings of sensitivity, resilience, and selfhood. These experiments are not confined to Japan; they resonate with global transformations in how mental health and vulnerability are conceived under neoliberal, pandemic, and now post-pandemic, strain. Terms like HSP are not simply adopted as truths; they are tested as practical vocabularies for living. Their value lies less in clinical accuracy than in social validity—in what they make articulable, recognizable, and potentially care-able. Each new term thus marks a small advance through which people bring the movements of body and mind, and their entanglement with “being in the world” (Merleau-Ponty, 1962), a little closer to shared understanding.

Yet within Japan’s idioms of endurance and adaptation, HSP acquires a distinctive moral resonance. It transforms fragility into a claim for recognition, redefining it as a means to remain responsive amid overwhelm. In the process, it does not reject resilience so much as make visible what resilience costs.

HSP is unlikely to be the last such term to rise, circulate, and fade within Japan’s wider ecology of everyday diagnostic labels. What shifts from one idiom to the next is not simply vocabulary, but the sociohistorical “ecological niche” (Hacking, 1999) in which certain forms of distress become newly recognizable—and newly speakable. For clinicians, this poses a familiar dilemma: how to respond to categories that may lack diagnostic standing, yet carry real explanatory and moral force for those who use them.

Post-pandemic, in a world that increasingly demands “resilience,” HSP—alongside its predecessors—offers a way to be affected without being disqualified. It makes the scars of overstimulation, exhaustion, and interpersonal pressure speakable not as evidence of immaturity, but as evidence that the world itself is asking too much.

## Data Availability

No datasets were generated or analyzed during the current study.

## Footnotes

1 In contrast to the asset framing central to HSP discourse in the United States and parts of Europe — the idea that sensitivity confers heightened empathy or creativity as professional and personal strengths — was largely absent from the conversations I observed. At least in the contexts I encountered, HSP was not deployed as a competitive advantage or marker of distinction but as an explanation for why things had always been difficult.

2 In my fieldwork, interlocutors did not claim HSP as an biological fact so much as a retrospective explanation: it accounted not just for present difficulty but for the entire arc of a life. To say "I am HSP" was less a claim about fixed temperament than a reinterpretation of personal history — the reason school had been hard, the reason work had felt unbearable, the reason one had always felt slightly out of step. HSP offered a coherent biographical narrative where previously there had been only accumulated evidence of personal failing.

3 **During 2020–2022, Japanese media attention to HSP surged:** publishers issued multiple new HSP titles (e.g., Iwanami Junior Shinsho’s *繊細すぎてしんどいあなたへ—HSP相談室* in **2020**; KADOKAWA’s Ryota/Cocoyowa self-help title in **2021**; Kanekoshobo’s research monograph *HSPの心理学* in **2022**), YouTube counselors hosted live “sensitivity” Q&As (e.g., Cocoyowa channel lives in **Jan–Feb 2022**), and NHK carried dedicated segments on HSP/HSC (e.g., **Nov 25, 2020** *ハートネットTV* “これだけは知ってほしい!“HSP”のこと”; **June 16, 2022** *きょうの健康* “HSP (とても繊細な人) ってなに?”; plus an **ETV特集** on HSC in **2020**).

4 For readers familiar with Japanese cultural idioms: this will likely recall *gaman* (endurance/self-restraint).

5 My last visit to Tokyo was in 2024 and was too brief to sustain fieldwork; in 2025, a Tokyo-based reviewer noted that they rarely heard HSP in everyday talk anymore. If so, the term’s rapid rise during the pandemic may also have been followed by a partial fading—an arc that underscores the social life of idioms of distress, which can intensify, circulate, and recede even as the suffering they name persists.

6 For example, see Allan Young’s work institutional construction of PTSD in U.S. psychiatry (1995), which argues PTSD was not simply the recognition of a preexisting natural kind, but the production of a diagnostic object that could stabilize experience, authorize treatment, and align suffering with clinical and bureaucratic infrastructures.

7 It’s important to reiterate that even Elaine Aron argued that HSP is not an illness or a diagnosis, simply a descriptor. .

## References

[CR2] Allison, A. (2013). *Precarious Japan*. Duke University Press.

[CR3] Aron, E. N. (1996). *The highly sensitive person: How to thrive when the world overwhelms you*. Broadway Books.

[CR4] Aron, E. N., & Aron, A. (1997). Sensory-processing sensitivity and its relation to introversion and emotionality. *Journal of Personality and Social Psychology,**73*(2), 345–368. 9248053 10.1037//0022-3514.73.2.345

[CR5] Asahi Shimbun. (2022, May 20). *「敏感で感受性強い人」とは 川越で21日、映画と講演で学ぶ /埼玉県* [What are “people who are sensitive and highly perceptive”? Learning through a film screening and lecture in Kawagoe on the 21st / Saitama Prefecture]. *朝日新聞* (朝刊), 埼玉全県・地域総合, pp. 20

[CR6] Asahi Shimbun. (2023, April 26). *感受性強い人、「HSP」ブームの弊害 心理学者の飯村周平さんに聞く* [People with high sensitivity: The harms of the “HSP” boom—interview with psychologist Shuhei Iimura]. *朝日新聞* (朝刊), くらし2, pp. 22

[CR7] Asuka Shinsha. (2020, December 15). *ついに50万部突破!激動の2020年に異例のベストセラーになった『「繊細さん」の本』とは?* [Finally surpassing 500,000 copies! What made The Sensitive Person Book an unusual bestseller in the turbulent year 2020]. PR Times. https://prtimes.jp/main/html/rd/p/000000043.000052297.html

[CR8] Bagatell, N. (2007). Orchestrating voices: Autism, identity, and the power of discourse. *Disability & Society,**22*(4), 413–426.

[CR10] Borovoy, A. (2005). *The too-good wife: Alcohol, codependency, and the politics of nurturance in postwar Japan*. University of California Press.

[CR11] Borovoy, A. (2008). Japan’s hidden youths: Mainstreaming the emotionally distressed in Japan. *Culture, Medicine & Psychiatry,**32*(4), 552–576. 18818992 10.1007/s11013-008-9106-2

[CR12] Bowker, G. C., & Star, S. L. (1999). *Sorting things out: Classification and its consequences*. MIT Press.

[CR13] Briggs, C. L., & Hallin, D. C. (2016). *Making health public: How news coverage is remaking media, medicine, and contemporary life*. Routledge.

[CR14] Brinton, M. C. (2011). *Lost in transition: Youth, work, and instability in postindustrial Japan*. Cambridge University Press.

[CR17] Das, V. (2007). *Life and words: Violence and the descent into the ordinary*. University of California Press.

[CR18] Dumit, J. (2004). *Picturing personhood: Brain scans and biomedical identity*. Princeton University Press.

[CR19] Eyal, G., Hart, B., Onculer, E., Oren, N., & Rossi, N. (2010). *The autism matrix: The social origins of the autism epidemic*. Polity Press.

[CR20] Good, B. (1994). *Medicine, rationality, and experience: An anthropological perspective.* Cambridge University Press.

[CR21] Hacking, I. (1998). *Mad travelers: Reflections on the reality of transient mental illnesses*. University of Virginia Press.

[CR22] Hacking, I. (1999). *The social construction of what?* Harvard University Press.

[CR23] Hacking, I. (2007). Kinds of people: Moving targets. *Proceedings of the British Academy,**151*, 285–318.

[CR26] Iimura, S. (2022). *HSP no shinrigaku: Kagakuteki konkyo kara rikai suru “sensaisa” to “ikizurasa”* [HSP psychology: Understanding “sensitivity” and “difficulty in living” from scientific evidence]. Kanekoshobo.

[CR27] Ishihara, T. (2015). *Learning from Tōjisha-Kenkyū: Mental health patients studying their difficulties with their peers.* University of Tokyo. https://www.researchgate.net/publication/283439463

[CR28] Ismail, R. (2020). New starts at New Start: Recovery and the work of *hikikomori*. *Transcultural Psychiatry,**57*(5), 698–709. 10.1177/1363461520958337 33076790

[CR29] Jenkins, J. H. (2014). *Extraordinary conditions: Culture and experience in mental illness.* University of California Press.

[CR31] Kaiser, B. N., & Kohrt, B. A. (2018). Introducing cultural concepts of distress into DSM-5 and ICD-11: Key lessons for transcultural psychiatry. *Transcultural Psychiatry,**55*(2), 231–256.

[CR32] Kaiser, B. N., & Weaver, L. J. (2019). Culture-bound syndromes, idioms of distress, and cultural concepts of distress: New directions for an old concept in psychological anthropology. *Transcultural Psychiatry,**56*(4), 589–598. 31347475 10.1177/1363461519862708 PMC6724704

[CR33] Kato, T., Kanba, S., & Teo, A. R. (2020). Hikikomori: Multidimensional understanding, assessment, and future international perspectives. *Psychiatry and Clinical Neurosciences,**74*(9), 506–518. 31148350 10.1111/pcn.12895

[CR34] Kato, T. A., Sartorius, N., & Shinfuku, N. (2024). Shifting the paradigm of social withdrawal: A new era of coexisting pathological and non-pathological hikikomori. *Current Opinion in Psychiatry,**37*(3), 177–184. 10.1097/YCO.0000000000000929 38415743 PMC10990035

[CR36] Kirmayer, L. J., & Sartorius, N. (2007). Cultural models and somatic syndromes. *Psychosomatic Medicine,**69*(9), 832–840. 18040090 10.1097/PSY.0b013e31815b002c

[CR37] Kitanaka, J. (2012). *Depression in Japan: Psychiatric cures for a society in distress.* Princeton University Press.

[CR38] Kitanaka, J., & Ayaya, S. (2023, June 12). *Japan’s radical alternative to psychiatric diagnosis*. Aeon. https://aeon.co/essays/japans-radical-alternative-to-psychiatric-diagnosis

[CR39] Kleinman, A. (1988). *The illness narratives: Suffering, healing, and the human condition*. Basic Books. 10.1097/ACM.0000000000001864 28952997

[CR40] Kleinman, A., Eisenberg, L., & Good, B. (1978). Culture, illness, and care: Clinical lessons from anthropologic and cross-cultural research. *Annals of Internal Medicine,**88*(2), 251–258. 626456 10.7326/0003-4819-88-2-251

[CR41] Kondō, Y. (2021, June 10). *「HSPブーム」現象を考える [Reflecting on the HSP Boom]*. *Gendai Business.* https://gendai.media/articles/-/84184

[CR44] Kyodo News. (2020, May 6). Japan’s hikikomori share wisdom for self-isolation during coronavirus. *Kyodo News Service.*

[CR45] Lakoff, A. (2005). *Pharmaceutical reason: Knowledge and value in global psychiatry*. Cambridge University Press.

[CR46] Lock, M. (1993). *Encounters with aging: Mythologies of menopause in Japan and North America*. University of California Press.

[CR47] Lock, M. (2013). *The Alzheimer conundrum: Entanglements of dementia and aging*. Princeton University Press.

[CR48] Lock, M., & Nguyen, V.-K. (2010). *An anthropology of biomedicine*. Wiley-Blackwell.

[CR49] Lupton, D. (2014a). The digital patient experience: Health apps, self-tracking, and digital health. *Social Theory & Health,**12*(3), 256–270.

[CR50] Lupton, D. (2014b). The commodification of patient opinion: The digital patient experience economy in the age of big data. *Sociology of Health & Illness,**36*(6), 856–869. 10.1111/1467-9566.12109 24443847

[CR51] Lupton, D. (2017). *The quantified self: A sociology of self-tracking*. Polity Press.

[CR53] Martin, E. (2007). *Bipolar expeditions: Mania and depression in American culture*. Princeton University Press.

[CR54] Mattingly, C. (2014). *Moral laboratories: Family peril and the struggle for a good life*. University of California Press.

[CR55] Matza, T. (2018). *Shock therapy: Psychology, precarity, and well-being in postsocialist Russia.* Duke University Press.

[CR56] McCurry, J. (2023, February 9). *Japan has 1.5 million social recluses as pandemic worsens isolation*. The Guardian*.*

[CR57] McCurry, J. (2018). Japan’s mental-health patients take research into their own hands. *The Lancet Psychiatry,**5*(5), 356. 10.1016/S2215-0366(18)30123-1

[CR58] Merleau-Ponty, M. (1962). *Phenomenology of perception* (C. Smith, Trans.). Routledge & Kegan Paul.

[CR59] Mol, A. (2002). *The body multiple: Ontology in medical practice. *Duke University Press.

[CR60] Myers, N. (2015). *Recovery’s edge: An ethnography of mental health care and moral agency.* Vanderbilt University Press.

[CR61] Nakamura, K. (2013). *A disability of the soul: An ethnography of schizophrenia and mental illness in contemporary Japan.* Cornell University Press.

[CR62] Navon, D. (2019). *Mobilizing mutations: Human genetics in the age of patient advocacy.* University of Chicago Press.

[CR63] Navon, D., & Eyal, G. (2016). Looping genomes: Diagnostic change and the genetic makeup of the autism population. *American Journal of Sociology,**121*(5), 1416–1471. 10.1086/684201 27092389

[CR64] Nguyen, V.-K. (2010). *The republic of therapy: Triage and sovereignty in West Africa’s time of AIDS.* Duke University Press.

[CR65] NHK. (2019, November 14). *HSPブーム**: **「敏感すぎる私」たちの苦悩と希望 [The HSP Boom: The Struggles and Hopes of “Overly Sensitive Me”]*. *NHK Close-Up Gendai*. https://www.nhk.or.jp/gendai/articles/4371/

[CR66] NHK Heart Net TV. (2020, November 25). *Tonari no Arai-san: Kore dake wa shitte hoshii! “HSP” no koto* [Neighbor Arai-san: “This much you should know! About HSP”]. NHK Educational TV.

[CR67] NHK ETV Tokushuu. (2020). *Binkan-kun tachi no natsu* [The summer of the sensitive kids]. NHK Educational TV.

[CR68] NHK Kyou no Kenkou. (2022, June 16). *HSP (Totemo sensai na hito) tte nani?* [What is an HSP (a very sensitive person)?]. NHK Educational TV.

[CR69] Nichter, M. (1981). Idioms of distress: Alternatives in the expression of psychosocial distress. *Culture, Medicine & Psychiatry,**5*(4), 379–408. 7326955 10.1007/BF00054782

[CR70] Nichter, M. (2010). Idioms of distress revisited. *Culture, Medicine & Psychiatry,**34*(2), 401–416. 20495999 10.1007/s11013-010-9179-6

[CR72] Rose, N. (2007). *The politics of life itself: Biomedicine, power, and subjectivity in the twenty-first century*. Princeton University Press.

[CR500] Rosenberg, C. E. (2006). Contested boundaries: Psychiatry, disease, and diagnosis. *Perspectives in Biology and Medicine, 49*(3), 407–424. 16960310 10.1353/pbm.2006.0046

[CR75] Stevens, C. (2013). *Disability in Japan*. Routledge.

[CR76] Takahashi, T. (2020). *Hikikomori in the time of COVID-19: Isolation within isolation*. Japan Times.

[CR77] Takeuchi, Y. (2018).* 「繊細さん」の本: 自分の心を守りながら生きる方法 [The book of “sensitive people”: How to live while protecting your heart]*. Asuka Shinsha.

[CR79] Tran, A. L. (2015). Rich sentiments and the cultural politics of emotion in postreform Ho Chi Minh City, Vietnam. *American Anthropologist,**117*(3), 480–492.

[CR80] Tsuboi, Y. (2023, June 12). Japan’s radical alternative to psychiatric diagnosis. *Aeon.* https://aeon.co/essays/japans-radical-alternative-to-psychiatric-diagnosis

[CR81] Wired Japan. (2021, March 30). Pandemic isolation and Japan’s hikikomori: From pathology to adaptation. *Wired Japan.*

[CR82] Yahoo Japan News. (2020, August 14). *Minna hikikomori ni natta: Korona to jishuku no seikatsu* [Everyone became a hikikomori: Life under COVID self-restraint]. *Yahoo Japan News.*

[CR83] Yergeau, M. (2018). *Authoring autism: On rhetoric and neurological queerness*. Duke University Press.

[CR86] Young, A. (1995). *The harmony of illusions: Inventing post-traumatic stress disorder.* Princeton University Press.

[CR87] Zhan, M. (2009). *Other-worldly: Making Chinese medicine through transnational frames.* Duke University Press.

[CR88] Zhang, L. (2020). *Anxious China: Inner revolution and politics of psychotherapy.* University of California Press.

[CR89] Iimura, S. (2023). HSPブームの功罪を問う [Questioning the merits and demerits of the HSP boom] (岩波ブックレット No. 1074). 岩波書店.

